# Mandibular Reconstruction With Fibula Flap and Dental Implants Through Virtual Surgical Planning and Three Different Techniques: Double-Barrel Flap, Implant Dynamic Navigation and CAD/CAM Mesh With Iliac Crest Graft

**DOI:** 10.3389/fonc.2021.719712

**Published:** 2021-10-05

**Authors:** Raúl Antúnez-Conde, José Ignacio Salmerón, Alberto Díez-Montiel, Marc Agea, Dafne Gascón, Ángela Sada, Ignacio Navarro Cuéllar, Manuel Tousidonis, Santiago Ochandiano, Gema Arenas, Carlos Navarro Cuéllar

**Affiliations:** Maxillofacial Surgery Department, Hospital General Universitario Gregorio Marañón, Madrid, Spain

**Keywords:** virtual surgical planning (VSP), 3D printing (3DP), CAD/CAM titanium mesh, mandibular reconstruction, STL models, implant dynamic navigation, double-barrel flap, iliac crest graft

## Abstract

**Introduction:**

Vertical discrepancy between the fibula flap and the native mandible results in difficult prosthetic rehabilitation. The aim of this study was to evaluate the outcomes of 3D reconstruction of the mandible in oncologic patients using three different techniques through virtual surgical planning (VSP), cutting guides, customized titanium mesh and plates with CAD/CAM technology, STL models and intraoperative dynamic navigation for implant placement. Material and methods

**Material and Methods:**

Three different techniques for mandibular reconstruction and implant rehabilitation were performed in 14 oncologic patients. Five patients (36%) underwent VSP, cutting guides, STL models and a customized double-barrel titanium plate with a double-barrel flap and immediate implants. In six patients (43%), VSP, STL models and a custom-made titanium mesh (CAD/CAM) for 3D reconstruction with iliac crest graft over a fibula flap with deferred dental implants were performed. Three patients (21%) underwent VSP with cutting guides and customized titanium plates for mandibular reconstruction and implant rehabilitation using intraoperative dynamic navigation was accomplished. Vertical bone reconstruction, peri-implant bone resorption, implant success rate, effects of radiotherapy in vertical reconstruction, bone resorption and implant failure, mastication, aesthetic result and dysphagia were evaluated.

**Results:**

Significant differences in bone growth between the double-barrel technique and iliac crest graft with titanium mesh technique were found (p<0.002). Regarding bone resorption, there were no significant differences between the techniques (p=0.11). 60 implants were placed with an osseointegration rate of 91.49%. Five implants were lost during the osseointegration period (8%). Peri-implant bone resorption was measured with a mean of 1.27 mm. There was no significant difference between the vertical gain technique used and implant survival (p>0.385). Implant survival rates were higher in non-irradiated patients (p<0.017). All patients were rehabilitated with a fixed implant-supported prosthesis reporting a regular diet (80%), normal swallowing (85.7%) and excellent aesthetic results.

**Conclusions:**

Multi-stage implementation of VSP, STL models and cutting guides, CAD/CAM technology, customized plates and in-house dynamic implant navigation for mandibular defects increases bone-to-bone contact, resolves vertical discrepancy and improves operative efficiency with reduced complication rates and minimal bone resorption. It provides accurate reconstruction that optimizes implant placement, thereby improving facial symmetry, aesthetics and function.

## Introduction

Mandibular defects in oncologic patients cause severe bone and soft tissue defects, with their consequent aesthetic and functional sequelae, and immediate mandibular reconstruction is mandatory. Mandibular segmental defects lead to malocclusion, mandibular deviation, TMJ alterations and soft tissue retraction (1). Retrusion of the lower third of the face occurs, especially in cases where the mandibulectomy includes the symphysis and mandibular body. In addition, there is significant lower lip ptosis and lip incompetence. When resection is located on the body of the mandible, facial asymmetry is evident, with soft tissue collapse on the affected side (2–4). If the tumor resection in the mandible includes the mandibular condyle, these sequelae are even more significant (1). In addition to lip incompetence, other disabling functional sequelae include salivary incontinence, difficulty in chewing, swallowing, and speech articulation. Patients undergoing mandibular resection who do not receive reconstruction present progressive deviation and retrusion towards the affected side, increasing the functional sequelae exposed (1, 2). In addition, vertical masticatory movements are replaced by oblique and diagonal movements directed by a single temporomandibular joint which, added to the limited lingual mobility in many cases, increases the patient’s difficulties in social interaction.

Advances in reconstructive surgical techniques in the head and neck area have allowed a comprehensive treatment and a complete surgical approach with a significant improvement in the aesthetic and functional reconstruction of these patients. Microsurgical techniques, virtual surgical planning (VSP), CAD-CAM (computer-aided design/computer-aided manufacturing) technology, intraoperative dynamic navigation and advanced implantology have improved significantly the comprehensive reconstruction of the oncologic patients with segmental mandibular defects (5–8).

Free flaps are considered the main reconstructive technique for segmental defects of the mandible and adjacent soft tissues (9). Among them, the fibula free flap, the scapular flap, and the iliac crest flap stand out. The fibula flap has been the flap of choice since it was described for mandibular reconstruction due to several advantages: great length of bone which allows reconstructions of defects longer than 10 cm (10), medullary and periosteal vascular supply, a long and anatomically constant vascular pedicle (11), large skin paddle for soft tissue reconstruction, bicortical bone (10) and the feasibility for dental rehabilitation with implant-supported or implant-retained prostheses (12). The bicortical bone is ideal in order to achieve primary stability of dental implants, it provides the possibility of a two-team approach and the morbidity of the donor area is relatively low (2, 13). However, the fibula flap does not provide sufficient height of bone to restore the native height of the mandible (3). The vertical discrepancy between the remnant mandible and the fibula flap results in a reduction of the vertical dimension of the lower third of the face with the consequent aesthetic and functional sequelae and difficulty in implant placement and prosthetic rehabilitation that may cause implant overloading and endanger both the functional and aesthetic long-term results (9). There are several surgical techniques to solve this problem through virtual surgical planning: a double-barrel flap with a double-barrel customized titanium plate and immediate implant placement, a CAD/CAM titanium mesh filled with iliac crest onlay graft over the fibula flap in a second stage procedure, and a vertical distraction osteogenesis of the fibula flap. To complete the mandibular reconstruction and the aesthetic and functional oral rehabilitation of these patients, the placement of dental implants needs to be optimal. Therefore, intraoperative navigation allows highly accurate and predictable results necessary in cases with altered and complex mandibular anatomy.

The aim of this study was to evaluate the outcomes of three-dimensional aesthetic and functional reconstruction of the mandible in oncologic patients using three different techniques through virtual surgical planning: 1) VSP, STL models and cutting guides for mandibular resection and reconstruction with a double-barrel free flap and a customized double-barrel titanium plate with immediate implant placement; 2) VSP, STL models and a CAD/CAM titanium mesh with iliac crest graft in a second surgical procedure with delayed placement of dental implants; 3) VSP with cutting guides for mandibular reconstruction and intraoperative dynamic navigation for implant placement in a delayed surgical procedure. An additional aim of this study was to compare the success rate of the implants and the bone resorption between irradiated and non-irradiated patients. The specific aims were: a) to compare the vertical reconstruction of the mandible; b) to compare the peri-implant bone resorption; c) to compare the implant failure between the different techniques; d) to compare the effects of radiotherapy in vertical reconstruction and bone resorption; e) to stablish the association between radiotherapy and implant failure; f) to evaluate mastication; g) to evaluate the aesthetic result and h) to evaluate the presence or absence of dysphagia. The aesthetic result, the mastication and dysphagia were evaluated 1 year after prosthetic rehabilitation. The aesthetic and functional results with deglutition and speech articulation were evaluated 1 year after prosthetic rehabilitation. The review of medical records and data collection and the subsequent analysis of the data collected is endorsed by the Hospital Ethics Committee at Gregorio Marañón General Hospital, Madrid, Spain.

## Materials and Methods

To address the research purpose, the investigators designed and implemented a retrospective study during a 5-year period (2015–2020). Fourteen patients with segmental mandibular defect due to their oncologic process and mandibular segmental reconstruction with implant rehabilitation were included in this study at Hospital General Universitario Gregorio Marañón, Madrid, Spain. The inclusion criteria were: 1) oncologic patients with segmental mandibulectomy reconstructed through VSP with double-barrel fibula free flap, double-barrel customized plate and immediate implant placement with surgical guides; 2) oncologic patients with segmental mandibulectomy reconstructed with fibula flap and iliac crest graft with customized titanium mesh through VSP, CAD/CAM technology and dental implants in a second surgical stage; 3) oncologic patients with segmental mandibulectomy reconstructed with fibula flap and implant rehabilitation through VSP and “in house” dynamic navigation in a second surgical procedure. Patients with previous history of radiotherapy or chemotherapy were excluded from this study.

Eight oncologic patients were diagnosed with ameloblastoma and six patients were diagnosed with squamous cell carcinoma. In all cases, MRI was performed prior to surgical planning to ensure the viability of the patients’ tibioperoneal vessels. In patients in which a skin paddle was necessary to reconstruct the soft tissue defect, the perforating vessels were marked on the skin, and the fibula flap was designed to include the perforators. Oncologic resection with clear margins was achieved in all patients through virtual surgical planning and cutting guides that improved the precision of the bone resection. Immediate reconstruction with fibula flap through VSP was performed in all patients. Patients were distributed into three groups according to the mandibular reconstruction and implant rehabilitation techniques employed.

In five patients (35%), the reconstruction technique used was the double-barrel fibula flap through virtual surgical planning, 3D printed models and a double-barrel customized titanium plate manufactured with a CNC milling machine (plate thickness: 2.0 mm with 1.5 mm in the upper extension and a 2.0 mm screw system) (KLS Martin). Immediate implant placement was accomplished through the 3D printed cutting guide This technique was performed in patients in which sufficient length of bone and length of vascular pedicle was available to allow an optimal reconstruction without compromise of microvascular anastomoses. The design of the peroneal cutting guide included the surgical guide for immediate implant placement (Ticare^®^, Valladolid, Spain).

In six patients (43%), a secondary 3D reconstruction of the mandible was accomplished through VSP, STL models and a custom-made titanium mesh (CAD/CAM) (Maffinter^®^, Madrid, Spain) with iliac crest graft and dental implants to enable both vertical and horizontal reconstruction of the fibula flap. This technique was performed in patients in which no sufficient length of bone was available to perform a double-barrel flap for complete reconstruction of the defect or in patients in which a previous single fibula flap was performed and a secondary 3D reconstruction of the fibula was needed prior to achieve an optimal prosthodontic rehabilitation with dental implants. In non-irradiated patients the second surgical stage was performed 4 months after initial surgery. In one irradiated patient the surgery was performed one year after the end of radiotherapy. A two-team approach was accomplished and a cortico-cancellous graft from the anterior superior iliac crest was harvested due to its thicker cortical layer. As it was a cortico-cancellous graft and not a microsurgical flap, no cutting guides were used, and the adaptation of the graft to the CAD/CAM titanium mesh was performed using a standard freehand procedure. A cervical approach was performed to expose the fibula flap and remove the osteosynthesis material. In this way, the cortico-cancellous graft was isolated from the oral cavity. In all patients cortico-cancellous graft was fixed using CAD/CAM titanium mesh, adapted and fixed to the remaining fibula. Six months later, the three dimensions of the mandible were evaluated by the Radiology Department through CT scan, providing relevant quantitative data regarding bone volume and bone resorption. The height of the graft was measured in both sagittal and coronal CT sequences, while the width of the graft was measured in the axial CT sequences. An intraoral approach was planned, the titanium mesh was removed and dental implants (Ticare^®^, Valladolid, Spain) were placed in all patients, who were subsequently rehabilitated with a fixed implant-supported prosthesis with the aim of achieving a comprehensive reconstruction, both aesthetic and functional.

Three patients (22%) underwent VSP with cutting guides and customized titanium plates with fibula flap with minimal vertical discrepancy that did not require complementary techniques. Rehabilitation with dental implants was performed using intraoperative dynamic navigation in a second surgical stage in patients previously reconstructed with fibula flap. The intraoperative dynamic navigation technique for implant placement is a recent technique in our department and, therefore, in patients in whom the vertical discrepancy between the fibula flap and the remnant mandible was not significant, implant placement was performed using an “in house” intraoperative dynamic navigation. Virtual 3D models of the jaw and surrounding tissues were generated from preoperative CT scan using 3D Slicer open-source software. The models were imported into a dental planning software and optimal position of the implants were determined. The virtual planning was transferred into the navigation software and a Polaris Spectra (NDI, Waterloo, Canada) optical tracking system was used to assess the real-time positioning of the surgical instruments. Tracked instruments included the drilling handpiece, and a pointer device in order to register the landmarks. The handpiece tracking was possible due to the attachment of a 3D printed adaptor specifically designed which included the optical markers. A 3D printed reference with optical markers was also designed in order to track the patients’ position during surgery. This printed reference was attached to the jaw using a silicone jig fitted on the teeth. These references were manufactured with polylactic acid using a 3D printer at the hospital’s FabLab. A software application was developed in 3D slicer showing the real-time position of the handpiece with respect to the preoperative images, 3D models and virtual planning. The handpiece tip position was recorded during drilling and the application enabled an accurate control of the drilling trajectory in order to achieve the virtual planning. Intraoperative measurements were performed to assess the position and angular deviation of the dental implants. Postoperative CT scan and panoramic radiographs were performed to evaluate the surgical outcomes.

The prosthetic rehabilitation of the oncologic patients was carried out in the Maxillofacial Surgery Department with fixed implant-supported prostheses with the aim of achieving the best aesthetic and functional reconstruction. During the follow-up period, the vertical gain obtained after surgery with each technique, the peri-implant bone resorption, the implant success rate and the effects of radiotherapy, were evaluated. Measurement of vertical bone gain was performed in all patients before implant placement. Esthetic assessment by the patients was performed in all groups to address scores in facial symmetry, facial scarring and facial projection, and the results were classified with scores 1 (poor), 2 (fair), and 3 (good). Functional outcomes such as mastication and deglutition were also evaluated. Dysphagia was reported as “yes” or “no”, and mastication was assessed in all groups, and results were classified with scores 1 (liquid-soft diet), and 2 (regular/unrestricted diet).

Statistical analysis: qualitative variables were expressed as frequencies and percentages. Quantitative values were expressed as mean +/- standard error of mean. Mann-Whitney’s tests were used to compare differences between groups of quantitative variables. Qualitative variables were compared using Chi-squared test. The statistical analysis was performed using the software SPSS 25.0. p value < 0.05 was considered statistically significant.

## Results

The mean follow-up time was 26 months (range 6-48 months) with no flap loss observed in any of the cases. The vertical discrepancy was solved, achieving in two patients a partial overcorrection of 112%. Fourteen patients with segmental mandibular defects were reconstructed with a free fibula flap. In all patients the origin of the defect was oncologic of which eight patients (57%) presented diagnosis of ameloblastoma and six patients (43%) of squamous cell carcinoma. In five patients (35%), mandibular reconstruction was performed in a single stage by means of VSP, double-barrel fibula flap, double-barrel customized titanium plate and immediate implant placement through surgical guides. Six patients (43%) underwent a second surgery with an iliac crest graft adapted according to the titanium meshes made specifically for each patient using CAD-CAM technology. Six months later, the titanium meshes were removed and dental implants were placed. In 3 patients (22%), the vertical dimension of a simple fibula flap was sufficient to provide a good aesthetic and functional result and implant placement was performed through VSP and dynamic navigation in a second surgical procedure. Therefore, 65% of patients required more than one surgical intervention for complete rehabilitation. The smallest mandibular segment was 6.3 cm and the largest was 16.4 cm, with a mean of 10.2 cm. In six patients, the fibula flap was exclusively a bone flap (43%) and in eight patients osseocutaneous flaps were harvested (57%). One flap required surgical revision due to partial thrombosis of one of the anastomosed veins in the immediate postoperative period.

## Virtual Surgical Planning for Double-Barrel Free Flap, Cutting Guides, Customized Double-Barrel Plate and Immediate Dental Implants

Patients reconstructed with double-barrel fibula flap had a mean age of 46.4. Four patients were male (80%) and one patient was female (20%). Two patients were diagnosed with squamous cell carcinoma, 2 patients with multicystic ameloblastoma and 1 desmoplastic ameloblastoma. Three patients (60%) did not receive postoperative radiotherapy and two patients were irradiated (40%). Vertical reconstruction was 27.8+/-0.5mm and bone resorption was 1.23+/-0.09mm. A total of 20 implants were immediately placed with a mean of 3.8+/-0.26 implants per patient. There were no major complications. In one patient there was intraoral exposure of the osteosynthesis material, which was treated conservatively without incident. This technique allowed reconstruction of the natural mandibular height. Two patients (40%) with 8 implants were irradiated with 60Gy. Twelve implants (60%) were placed in the 3 non-irradiated patients. Of the implants placed in irradiated patients, one implant (12.5%) presented osseointegration failure. In non-irradiated patients there was loss of one implant (8.3%). The rest of the implants showed correct osseointegration (90%). All patients were rehabilitated with fixed implant-supported prostheses. One year after prosthetic rehabilitation, mesial and distal peri-implant bone resorption was evaluated. The mean bone resorption was 1.23 mm. In irradiated patients the resorption was slightly higher than in non-irradiated patients, where it was not relevant. (**Table 1A**).

**Table 1:** Outcomes with three different techniques for mandible reconstruction and implant rehabilitation in oncologic patients.

A.- DOUBLE BARREL FIBULA FLAP WITH INMEDIATE DENTAL IMPLANTS											
Gender/Age (Years)	Diagnosis	Length of Defect (cm)	Vertical Reconstruction (mm)	Vertical Fibula Height (mm)	Horizontal Dimension (mm)	Number ofImplants/ Failure	Bone resorption (mm)	Radiotherapy	Aesthetic Result	Mastication	Dysphagia
M/70	Squamous cell carcinoma	8.4	26.1	12.3	8.6	4/1	1.6	Yes	3	2	Yes
F/32	Ameloblastoma	7.8	24.5	12.0	8.0	3/1	1.5	No	3	2	No
M/29	Ameloblastoma	9.9	30.7	14.5	10.7	5	0.5	No	3	2	No
M/43	Ameloblastoma	9.3	28.2	13.7	9.5	4	1.2	No	2	2	No
M/58	Squamous cell carcinoma	10.6	29.4	14.2	10.9	4	1.4	Yes	3	1	No
Average		9.2	27.8	13.32	9.54	20/2 (90.0%)	1.23				
**B.- FIBULA FLAP WITH ILIAC CREST GRAFT, TITANIUM MESH AND DENTAL IMPLANTS.**											
**Gender/Age** **(Years)**	**Diagnosis**	**Length of Defect** **(cm)**	**Vertical Reconstruction** **(graft) (mm)**	**Vertical Fibula Height** **(mm)**	**Horizontal** **Dimension** **(mm)**	**Number of** **Implants/ Failure**	**Bone resorption** **(mm)**	**Radiotherapy**	**Aesthetic** **Result**	**Mastication**	**Dysphagia**
M/42	Ameloblastoma	9.5	11.7	14.1	11.2	4	1.5	No	3	2	No
F/61	Squamous cell carcinoma	10.8	11.9	12.3	9.6	4	1.5	No	2	1	No
F/63	Squamous cell carcinoma	9.6	12.3	12.1	9.4	5/1	1.6	No	2	2	No
M/38	Ameloblastoma	12.6	13.2	14.4	11.7	7	0.5	No	3	2	No
F/35	Ameloblastoma	9.4	13.4	13.9	9.7	4	1.4	No	3	2	No
M/73	Squamous cell carcinoma	8.9	10.1	14.2	8.9	4/1	2.4	Yes	3	2	Yes
Average		10.05	12.22	13.55	10.1	28/2 (92.86%)	1.48				
**C.- DYNAMIC NAVIGATION FOR IMPLANT REHABILITATION IN FIBULA FLAP**											
**Gender/Age** **(Years)**	**Diagnosis**	**Length of Defect** **(cm)**	**Vertical Reconstruction** **(mm)**	**Vertical Fibula Height** **(mm)**	**Horizontal** **Dimension** **(mm)**	**Number of** **Implants/ Failure**	**Bone resorption** **(mm)**	**Radiotherapy**	**Aesthetic** **Result**	**Mastication**	**Dysphagia**
M/18	Ameloblastoma	9.8	Not necessary	12.2	8.5	3	0.7	No	3	2	No
M/53	Ameloblastoma	8.8	Not necessary	13	9.2	4	0.8	No	3	2	No
M/61	Squamous cell carcinoma	8.2	Not necessary	11.8	9.8	5/1	1.8	Yes	2	2	No
Average		8.9		12.3	9.2	12/1 (91.6%)	1.1				

Esthetic result: 3=good; 2= fair; 1=poor. Mastication: 2= unrestricted diet; 1 = liquid and soft diet.

### Case Presentation

**Figure 1:**
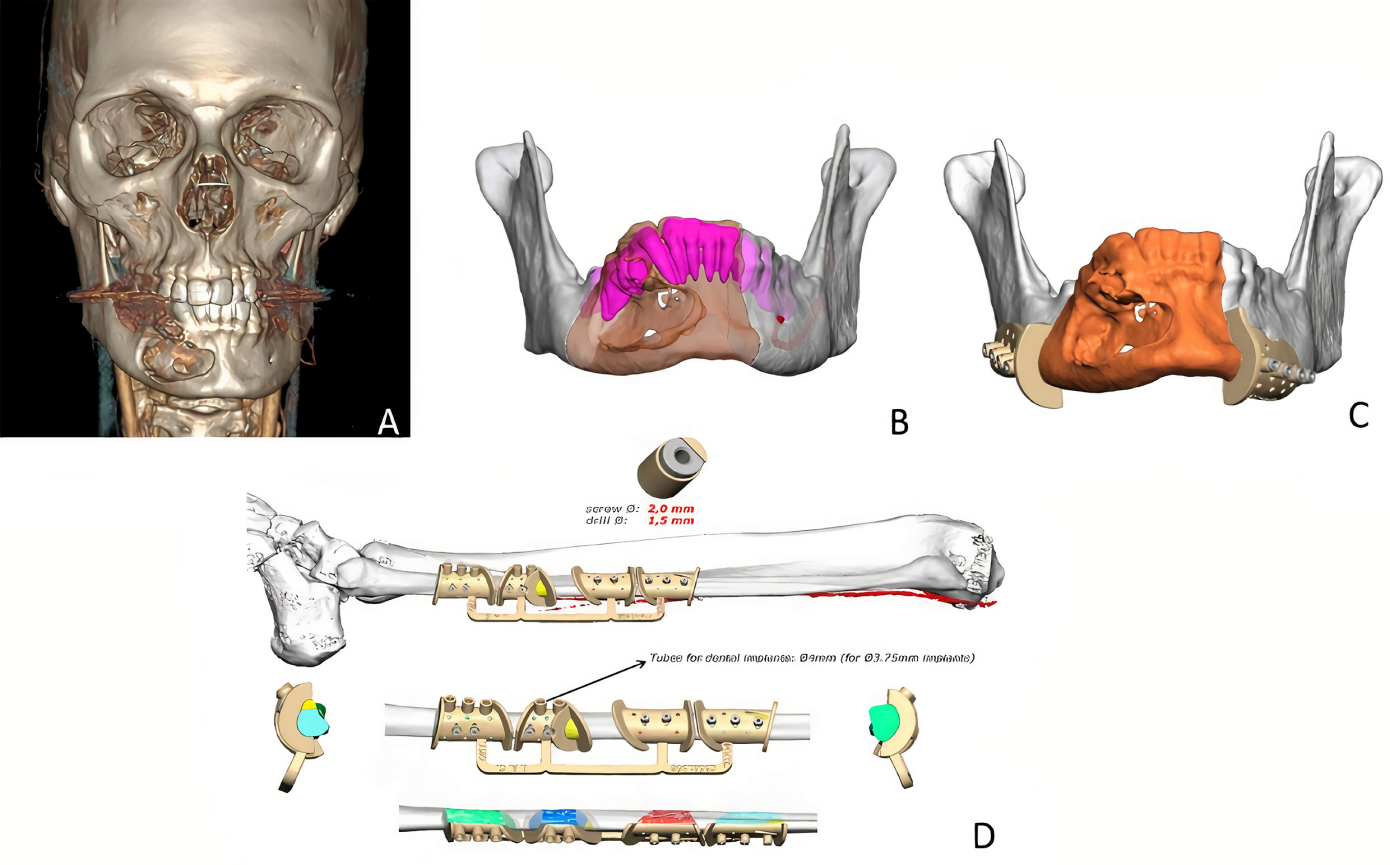
**(A)** CT scan showing a right ameloblastoma. **(B)** Virtual planning of the mandibular resection. **(C)** Cutting guides designed for tumor resection with clear margins. **(D)** Fibula cutting guide designed for a double-barrel flap in 4 segments with the implant slots for implant placement.

**Figure 2:**
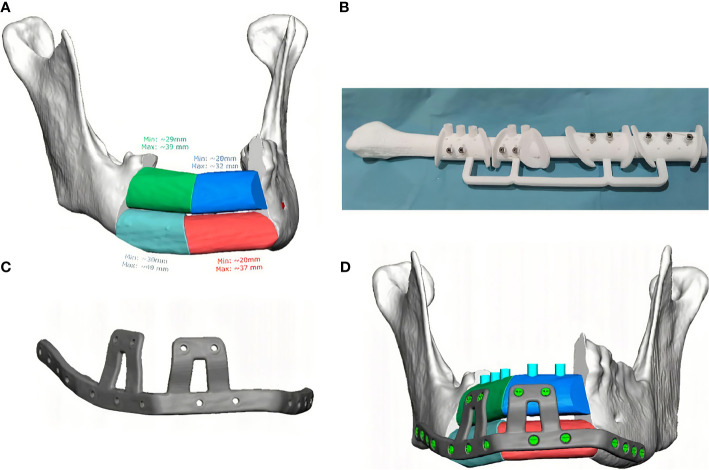
**(A)** Double-barrel fibula flap designed in four segments for 3D reconstruction of the mandible. **(B)** Fibula cutting guide including the slots for implant placement. **(C)** Customized double-barrel titanium plate for rigid fixation. **(D)** Final design for mandibular reconstruction including the double-barrel flap, the double-barrel titanium plate and the position of dental implants.

**Figure 3:**
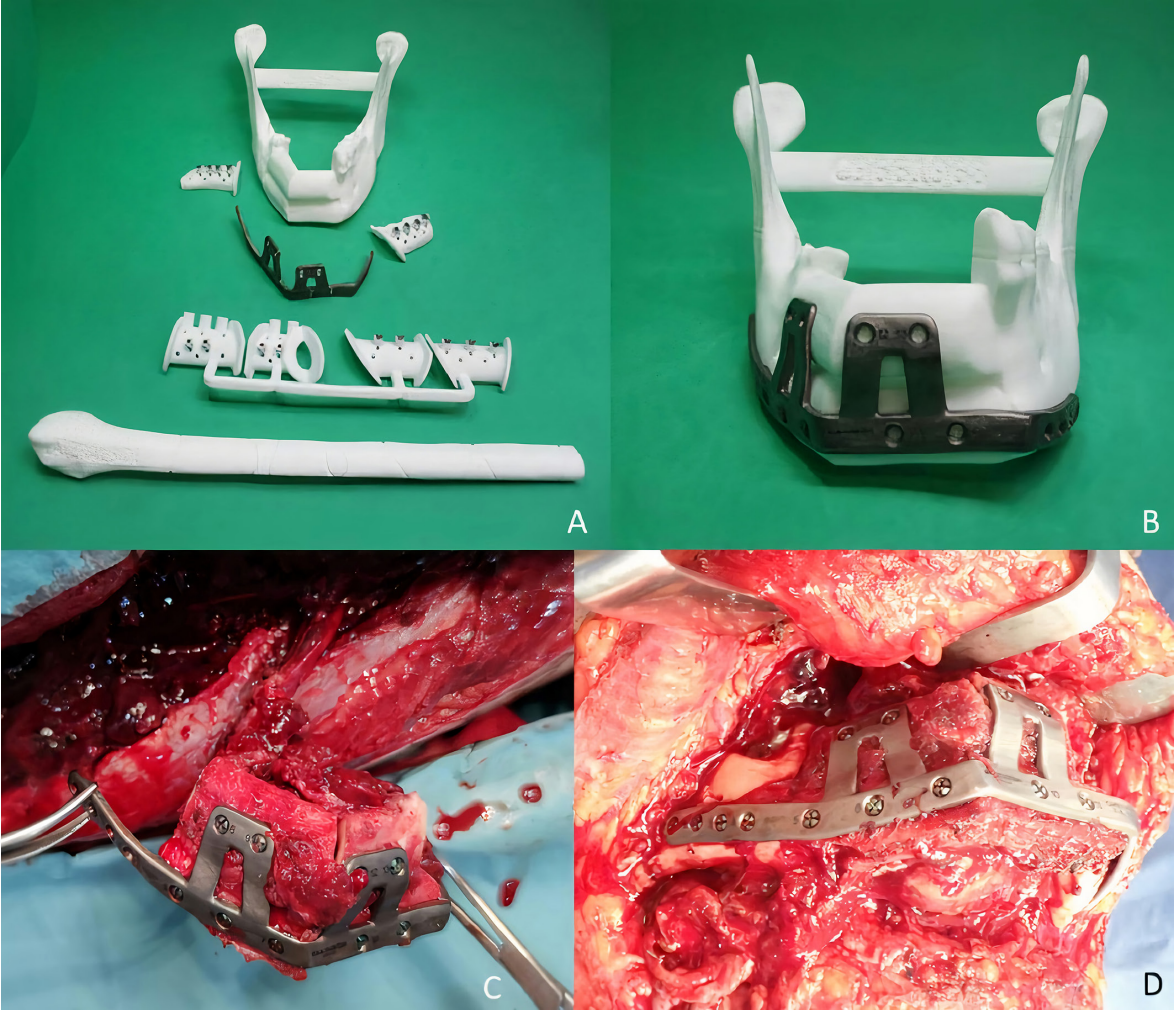
**(A, B)** STL printed models with cutting guides and customized titanium plate. **(C)** Double-barrel fibula flap fixed to the titanium plate prior to vascular pedicle clamping. **(D)** Mandibular reconstruction after microvascular anastomoses.

**Figure 4:**
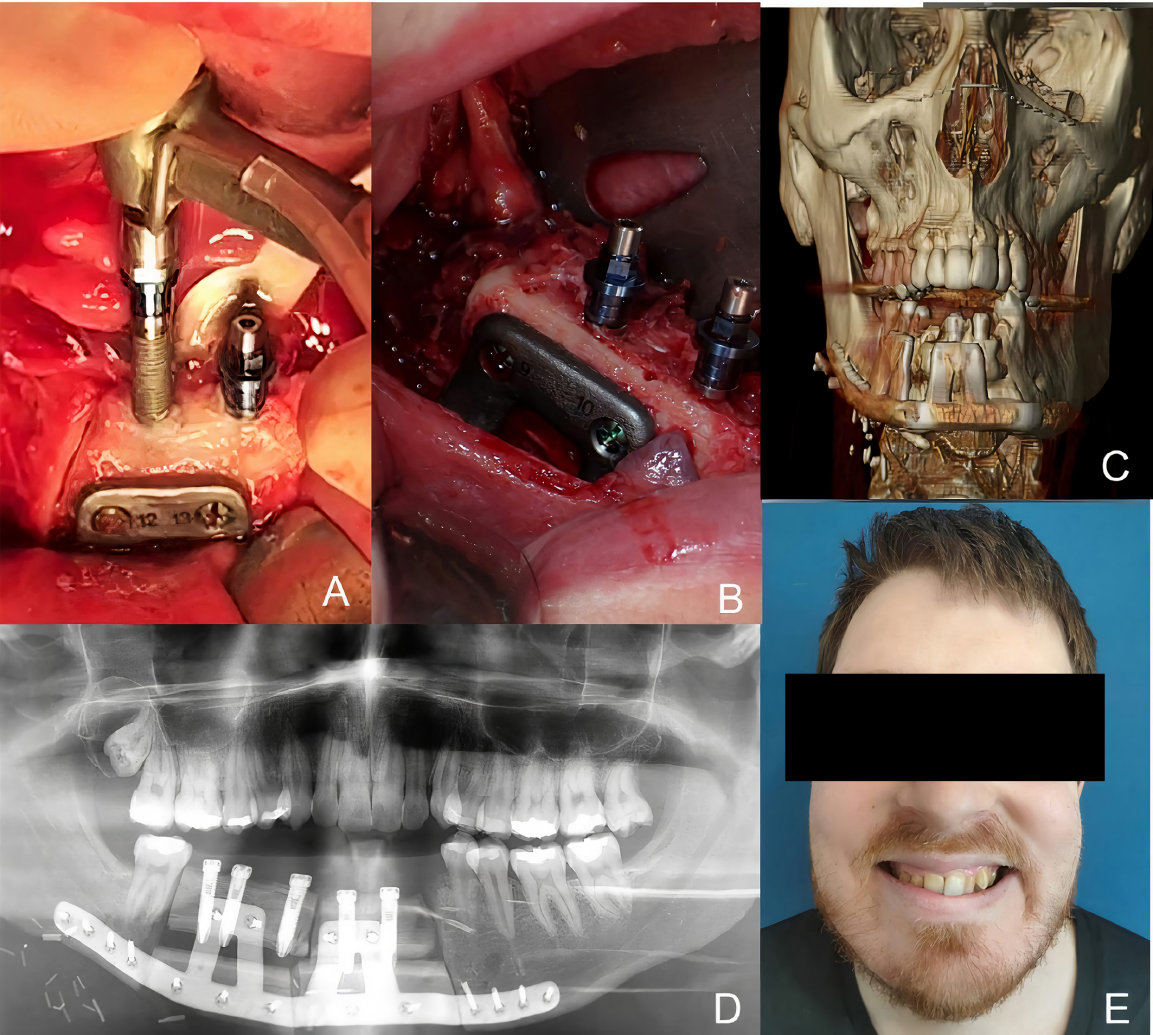
**(A, B)** Immediate implant placement between the osteosynthesis material as previously planned in the VSP. **(C)** Postoperative CT scan demonstrating the 3D reconstruction with the double-barrel free flap, the double-barrel customized plate and the immediate implants. **(D)** Panoramic radiograph after surgery with 3D reconstruction of the mandible. **(E)** Final aesthetic and functional result.

A 29-year-old male patient was diagnosed with mandibular desmoplastic ameloblastoma with involvement of the symphysis and right mandibular body and disruption of the lingual cortical bone. A virtual surgical planning was performed for resection with clear margins and reconstruction of a double-barrel fibula flap with four segments and immediate placement of five dental implants were performed (**Figure 1**). Cutting guides were designed for both resection and reconstruction, as well as a design for immediate implant placement (Osteoplac Innovations^®^, Madrid, Spain). For the reconstruction, a customized double-barrel titanium plate was designed and manufactured with a CNC milling machine (plate thickness: 2.0 mm with 1.5 mm in the upper extension and a 2.0 mm screw system) (KLS Martin) specifically so that the placement of the implants did not interfere with the osteosynthesis material (**Figure 2**). A two team approach was performed simultaneously and the cutting guides were used to perform the segmental mandibulectomy and to perform the osteotomies for a double-barrel fibula flap with four segments. The fibula cutting guide incorporated in its design the slots for the placement of the implants that were drilled during the harvesting of the flap. Once the mandible had been reconstructed and the microsurgical anastomoses had been performed, five dental implants were placed (Ticare^®^) (**Figures 3**, **4**). Postoperative CT scan and panoramic radiograph demonstrated a correct union between the fibula flap and the remnant mandible and accurate implant placement as planned in the virtual surgical planning. Prosthetic rehabilitation was performed with an implant fixed prosthesis with an optimal aesthetic and functional result (**Figure 4**).

## Virtual Surgical Planning, Stereolitographic Models and CAD/CAM Titanium Mesh for 3D Reconstruction of Fibula Flap With Iliac Crest Graft and Dental Implants

The patients reconstructed with fibula flap and titanium mesh with iliac crest graft had a mean age of 52 years. Three patients were female (50%) and three patients were male (50%). Three patients were diagnosed with squamous cell carcinoma, 1 patient developed a recurrent ameloblastoma and 2 patients were diagnosed with multicystic ameloblastoma. One patient (17%) received postoperative radiotherapy while 5 patients (83%) were not irradiated. Mean vertical reconstruction was 12.1 mm (range 13.4 - 10.1 mm). Mean bone resorption was 1.48 mm. One patient received radiotherapy and iliac crest graft was delayed one year after the end of radiotherapy. In the remaining patients (83%), reconstruction of the vertical dimension with iliac crest graft and custom CAD-CAM titanium mesh was performed four months after initial surgery. In all cases a two-team approach was performed, at the cervical area and the anterosuperior iliac spine, respectively. The cervical approach allowed placement of the iliac crest graft without contamination of the oral cavity in all cases. Six months later, an intraoral approach was performed for mesh removal and implant placement. Twenty-eight implants were inserted in this group of patients with an average of 4.6 implants per patient. Osseointegration failure was evident in one implant (3.5%) in the irradiated patient. The osseointegration success rate was 92.86%. All patients were rehabilitated with an implant-supported fixed prosthesis (**Table 1B**).

### Case Presentation

**Figure 5:**
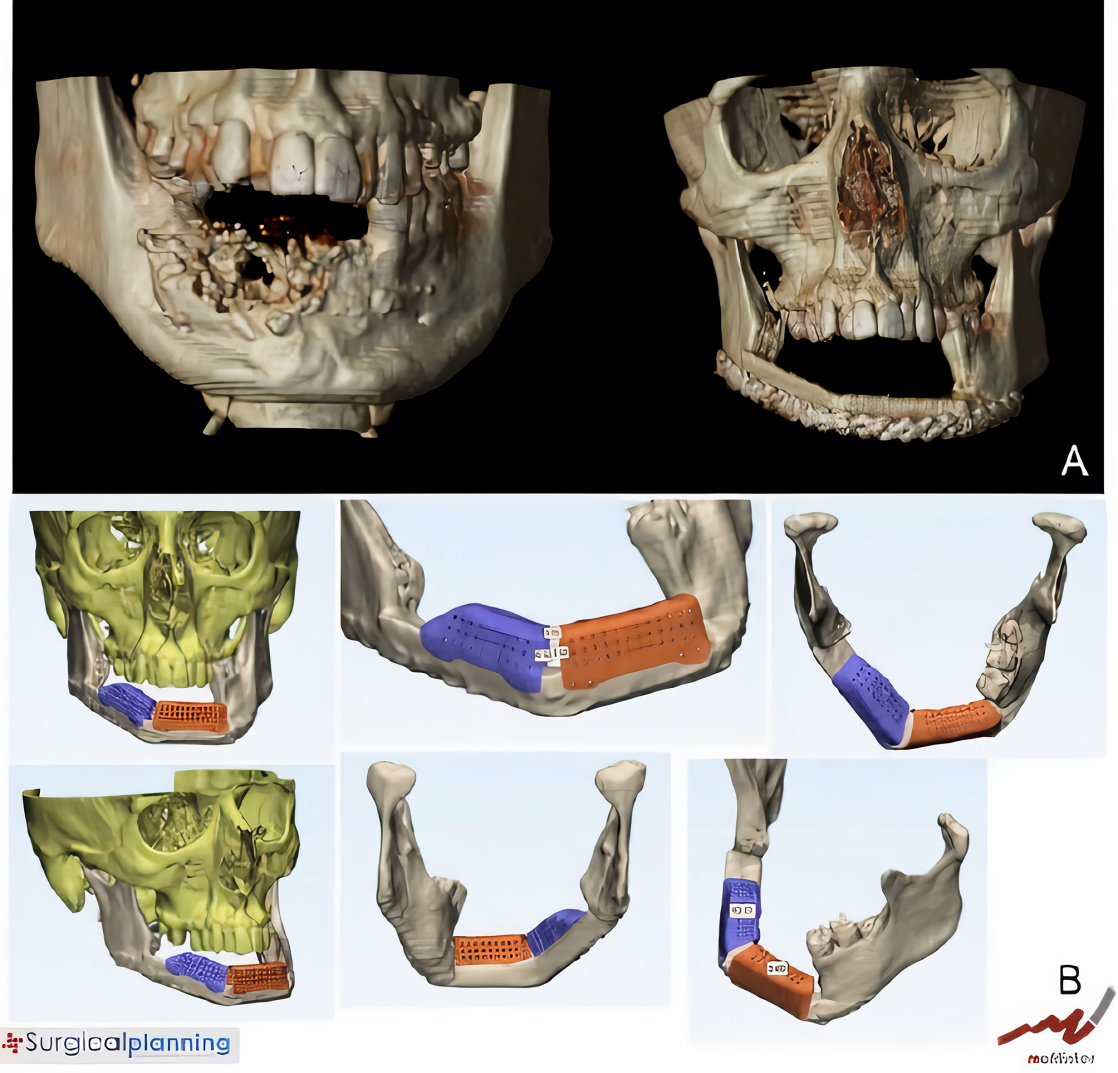
**(A)** CT Scan showing destruction of the mandibular symphysis, segmental mandibulectomy from left mandibular body to right mandibular angle and immediate reconstruction with fibula free flap. **(B)** VSP for vertical reconstruction of the fibula with two CAD/CAM titanium meshes adapted to the two segments of the fibula flap.

**Figure 6:**
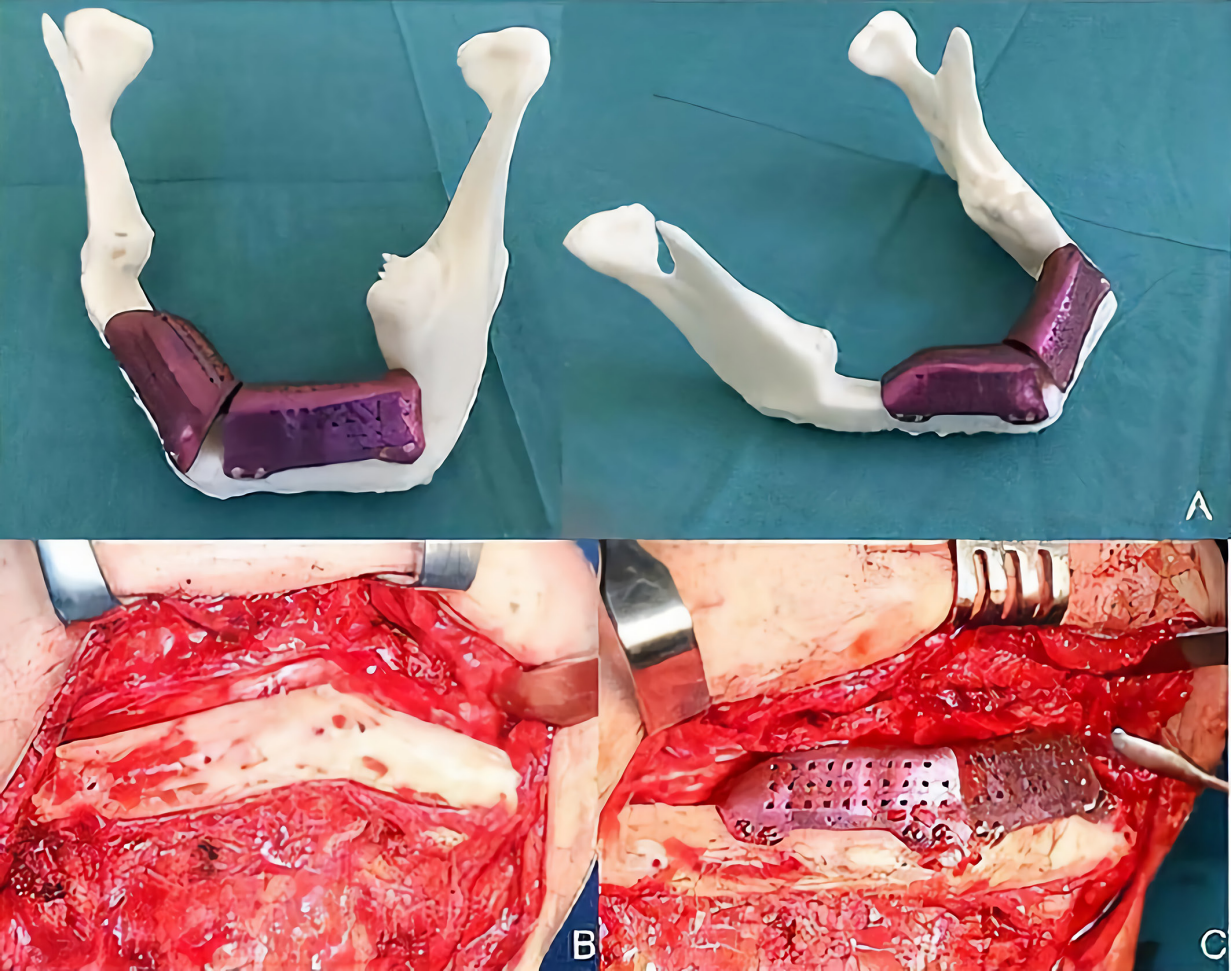
**(A)** (STL) showing the vertical bone discrepancy and the adaptation of CAD/CAM titanium mesh to the fibula for three-dimensional bone reconstruction. **(B)** Cervical approach to avoid intraoral communication and exposure of the peroneal flap with good bone vascularization. **(C)** Iliac crest cortico-cancellous graft to reconstruct mandibular height and preserve the horizontal dimension of the fibula. Adjustment of the titanium mesh to the upper and lateral part of the two fibula segments.

**Figure 7:**
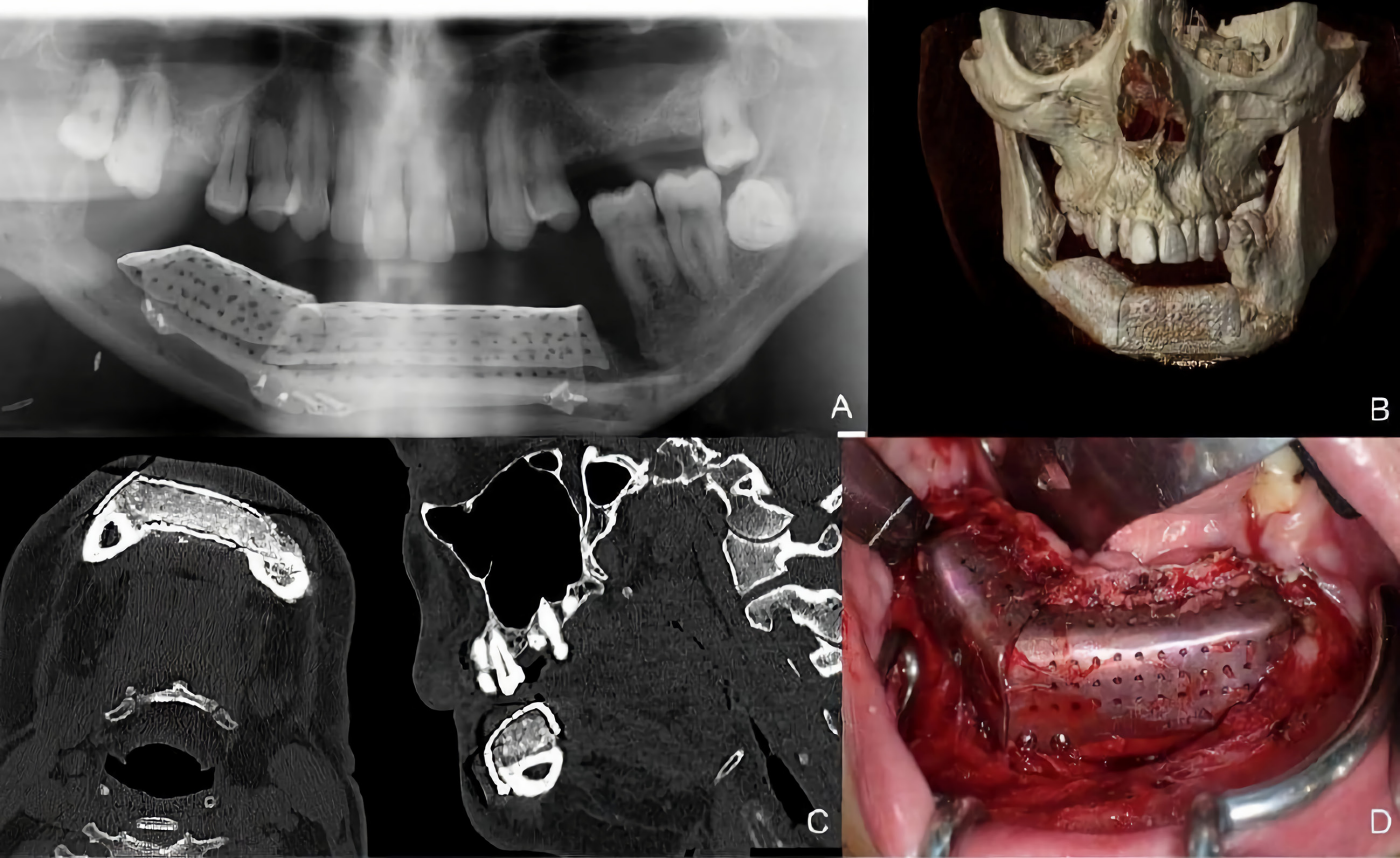
**(A)** Panoramic radiograph showing a bone gap between the iliac crest graft and the remnant mandible mesially to the lower molar due to the angular shape of the titanium mesh at this level. CT Scan **(B)** with three-dimensional reconstruction of the mandible. CT Scan demonstrating the stability of the transverse dimension of the fibula with respect to the remnant mandible. **(C)** The three-dimensional preservation of the iliac crest graft with CAD/CAM mesh makes it possible to double the height of the fibula. **(D)** Intraoral approach showing the three-dimensional preservation of the customized mesh.

**Figure 8:**
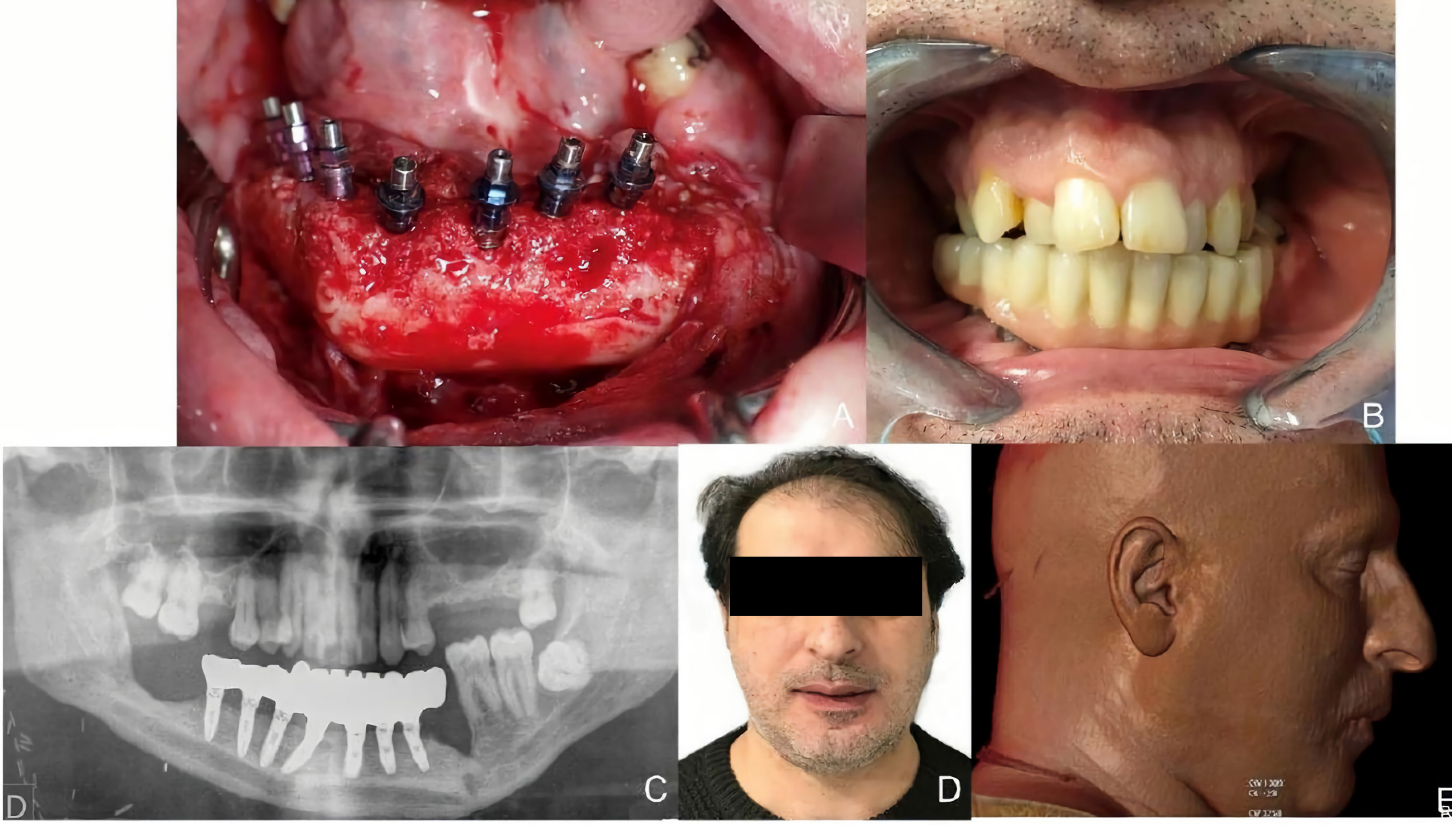
**(A)** Placement of Ticare^®^ dental implants. **(B)** Final dental restoration. **(C)** Panoramic radiograph demonstrating the reconstruction of the previous height of the mandible with a correct osseointegration of the implants. **(D)** Aesthetic result with mandibular symmetry. **(E)** Morphing performed after implant rehabilitation, demonstrating a significant aesthetic improvement after 3D reconstruction with iliac crest graft.

A 38-year-old patient was referred to our Department reporting progressive deformity of the mandibular symphysis. 3D CT scan showed a lytic lesion with destruction of the external mandibular cortex (**Figure 5A**). A multicystic ameloblastoma was diagnosed. Tumor resection with segmental mandibulectomy and clear margins, and immediate reconstruction with a two-segment fibula flap was performed (**Figure 5A**). A vertical discrepancy between the remnant mandible and the fibula flap was assessed and a virtual surgical planning (VSP) with a cortico-cancellous iliac crest graft was planned in a second surgical procedure. VSP was performed with the biomedical engineer (Maffinter^®^, Madrid, Spain) and a three-dimensional virtual reconstruction of the defect was performed with two titanium CAD/CAM meshes (**Figure 5B**). STL model and CAD/CAM titanium mesh were printed and checked before surgery (**Figure 6A**). Under general anesthesia, a cervical approach was performed to expose the fibula and remove the osteosynthesis material without communicating with the oral cavity (**Figure 6B**). Simultaneously, a cortico-cancellous graft of the left anterosuperior iliac crest was obtained. The graft was fixed to the fibula using the CAD/CAM titanium mesh and 1.5 mm screws (**Figure 6C**). There was no intraoral exposure of the graft and an increase in the vertical dimension of the fibula was achieved and demonstrated by panoramic radiograph and CT scan. Panoramic radiograph revealed a bone gap between the iliac crest graft and the remnant mandible mesial to the left first molar due to the angular shape of the titanium mesh at this level. Six months later, the ossification of the graft and volume of the soft tissue were verified. An intraoral approach was performed, the titanium mesh was removed and the increase in height and width achieved with the graft was verified, showing the space beneath the titanium mesh completely filled with new hard tissue (**Figure 7**). Seven dental implants were placed (Ticare^®^, Valladolid, Spain) and, four months later, the second surgical procedure of the implants was performed. Despite the gap between the iliac crest graft and the remnant mandible, the prosthetic rehabilitation was carried out by means of a fixed implant-supported prosthesis providing normal occlusion (**Figure 8**). Three years later, there was no evidence of significant peri-implant bone resorption. The prosthetic rehabilitation allowed a correct aesthetic and functional result with a regular diet and intelligible speech.

## Virtual Surgical Planning and In-House Implant Dynamic Navigation for Mandibular Reconstruction and Dental Rehabilitation

In three cases (21%), reconstruction with a free fibula flap was sufficient to obtain a vertical dimension of the segmental defect similar to the remnant mandible, and no further techniques for vertical reconstruction were needed. In patients reconstructed with simple fibula flap, we usually deferred the placement of the implants because the possible interference of the osteosynthesis material could lead us to place the implants in a non-desirable position for future prosthetic rehabilitation. Therefore, implant dynamic navigation was performed to place dental implants in patients previously reconstructed with a simple fibula flap in whom implants had not been placed in the first surgical procedure. Implant rehabilitation of these patients was performed using 3D customized splint and intraoperative dynamic navigation. The mean age of these patients was 43 years. One patient was diagnosed with squamous cell carcinoma and 2 patients were diagnosed with ameloblastoma. Two patients (67%) did not receive radiotherapy, and implant surgery was performed four months after mandibular reconstruction. One patient (33%) was irradiated after surgery and implant placement was performed one year after completion of radiotherapy. Twelve implants were placed assisted by this technology, of which seven (58.3%) were placed on non-irradiated peroneal flap and five (41.6%) on the irradiated patient. The average deviation in implant position was less than 1 mm and the average deviation in implant angulation was less than 5°. No major complications occurred. The mean peri-implant bone resorption measured in mesial and distal locations was 1.1 mm. One implant loss occurred in the irradiated patient, with a success rate of 91.6% (**Table 1C**).

### Case Presentation

**Figure 9:**
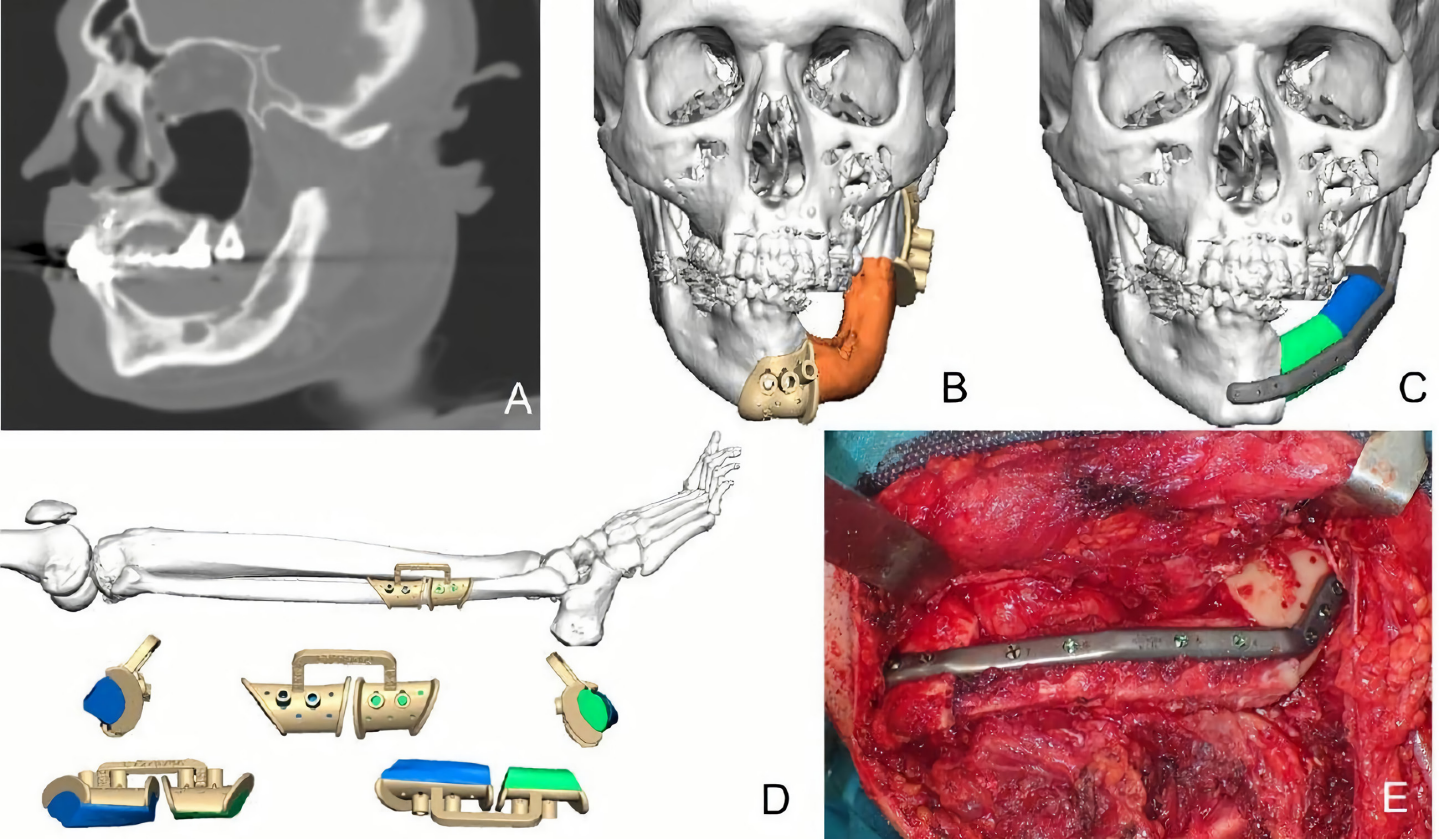
**(A)** Recurrent ameloblastoma. **(B)** Virtual surgical planning of the oncologic resection with cutting guides. **(C)** Virtual reconstruction with fibula free flap and customized titanium plate. **(D)** Cutting guides for fibula flap. **(E)** Immediate reconstruction with fibula flap and customized titanium plate.

**Figure 10:**
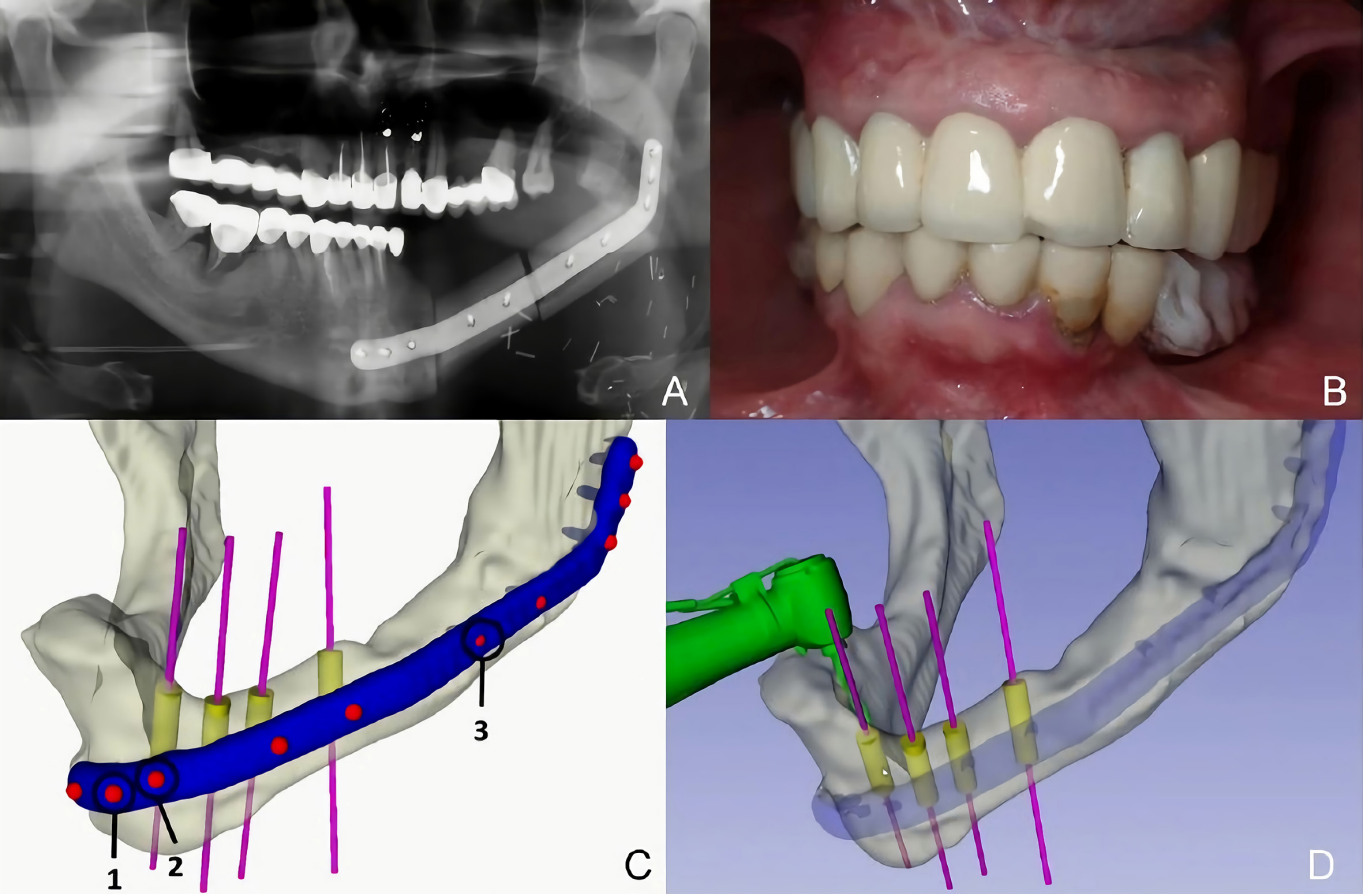
**(A)** Panoramic radiograph after mandibular reconstruction. **(B)** Splint manufactured for dynamic navigation. **(C)** Virtual surgical planning of 4 Ticare^®^ implants. **(D)** Intraoperative dynamic in-house navigation for implant placement.

**Figure 11:**
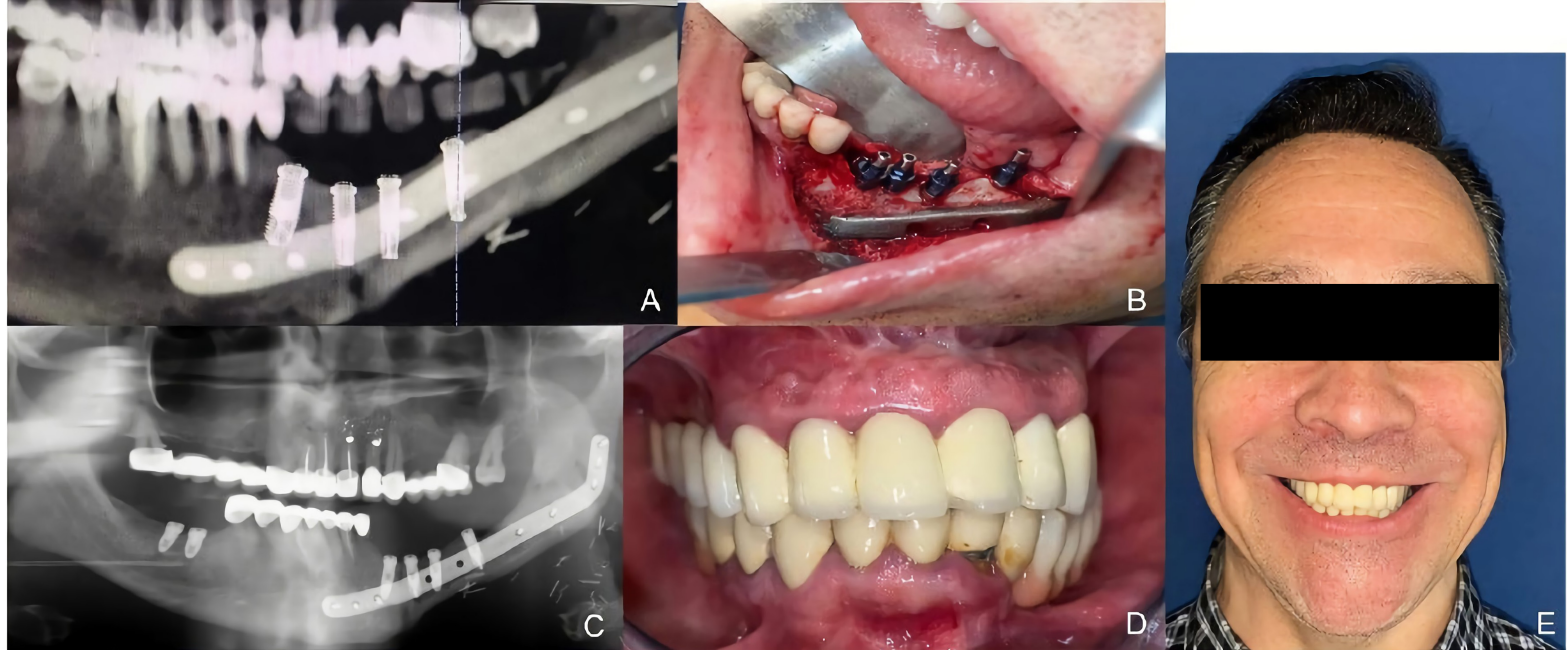
**(A)** Virtual planning of dental implants. **(B)** Intraoperative implant placement through dynamic navigation. **(C)** Panoramic radiograph after surgery showing a high accuracy compared with the virtual planning. **(D)** Prosthetic rehabilitation with implant fixed prosthesis. **(E)** Aesthetic and functional result.

A 53-year-old patient was diagnosed with a recurrent left mandibular ameloblastoma. A virtual surgical planning of a segmental mandibulectomy from the parasymphysis to the left subcondylar area was performed, with the corresponding cutting guides designed. Virtual planning of mandibular reconstruction with fibula flap and customized titanium plate was designed simultaneously with the cutting guides for the fibula flap to perform the osteotomies. The patient underwent segmental mandibulectomy with clear margins through the printed cutting guides and immediate reconstruction with a fibula free flap and a customized titanium plate. In the control panoramic radiograph, the mandibular reconstruction showed the mandibular reconstruction carried out by means of virtual surgical planning (**Figure 9**). Four months later, when planning the implant placement, it was decided to perform a dynamic intraoperative navigation. Splints were manufactured for CT scan and navigation planning of Ticare^®^ implant placement. The placement of the implants was planned by means of in-house dynamic navigation using the 3D Slicer software (**Figure 10**). The “In House” navigation system uses an optical system with two infrared cameras and a free 3D Slicer software. The navigator detects the reflective spheres placed on the pointer, the handpiece adapter and the silicone key kit that is used as intraoral reference. This kit allows the navigator to compensate mandibular movements with submillimetric precision. The Ticare^®^ implants were placed in the fibula flap using dynamic navigation with a mean deviation of less than 1 mm and an angular deviation of less than 5°. In the comparison between the preoperative planning of the implants and the postoperative panoramic radiograph after implant placement using “in house” navigation, it was observed that the accuracy achieved was submillimetric (**Figure 11**). Prosthetic rehabilitation was performed with an implant supported prosthesis and aesthetic and functional results were reported as excellent.

#### Vertical Mandible Reconstruction and Bone Resorption

Using Mann-Whitney test, the authors demonstrated significant differences in bone vertical gain in millimeters between the double barrel technique and iliac crest graft with titanium mesh technique (p<0.002). Regarding bone resorption in millimeters after implant placement and loading, the Mann-Whitney test determined that there were no significant differences between the double-barrel technique and the iliac crest graft technique (p=0.11).

#### Implant Success Rate

The overall implant success rate was 91.49%. By groups, the success rate of immediate implants on double-barrel fibula flap was 90%, 92.86% on iliac crest graft and 91.6% on peroneal flap with intraoperative navigation. Statistical analysis using the Chi-square table showed no statistically significant differences between the vertical gain (mm.) technique used and implant survival (p>0.385) (**Table 2**).

**Table 2:** Implant failure between the different techniques.

			Implant Failure		*p* Value
			NO *n *(%)	YES *n *(%)	
TECHNIQUE	Double barrel fibula flap	Rate	18 (30%)	2 (3.3%)	0.385
	Iliac crest graft with titanium mesh over fibula flap	Rate	26 (43.3%)	2 (3.3%)	
	Dynamic navigation for implants in fibula flap	Rate	11 (18.3%)	1 (1.6%)	

#### Effect of Radiotherapy in Vertical Reconstruction and Bone Resorption 

The Mann-Whitney test was applied to compare bone height and bone resorption in patients submitted to radiotherapy and showed no statistically significant difference, and radiotherapy was not associated with higher bone resorption (**Table 3**).

**Table 3:** Effects of radiotherapy in vertical reconstruction and bone resorption.

	Radiotherapy	No Radiotherapy	*p*
Vertical Reconstruction (mm)	16.5+/-1.25	16.21+/-0.74	0.22
Bone resorption (mm)	1.8+/-0.21	1.12+/-0.10	0.26

#### Association Between Radiotherapy and Implant Failure 

Of the 60 implants, 43 implants were placed in non-irradiated bone with two losses (4.6%) and a success rate of 95.4%. Seventeen implants were inserted in irradiated bone, with two failures of osseointegration (11.7%) and a success rate of 88.3%. Chi-square analysis demonstrated significant results between both groups (p<0.017) (**Table 4**).

**Table 4:** Association between radiotherapy and implant failure.

			Implant Failure		*p* Value
			NO *n *(%)	YES *n *(%)	
Radiotherapy	NO	Rate	41 (68.3%)	2 (3.33%)	0.017
	YES	Rate	14 (23.3%)	3 (5.0%)	

### Mastication

Mastication was assessed in all groups, and results were classified with scores 1 (liquid-soft diet), and 2 (regular/unrestricted diet). Four patients reconstructed with a double-barrel flap and dental implants reported an unrestricted diet (80%), while 1 patient referred a soft diet (20%). Five patients reconstructed with a titanium mesh, iliac crest graft and implants reported a regular diet (83.3%), while 1 patient referred a liquid diet (16.7%). All patients in which oral rehabilitation was performed through dynamic navigation reported a regular diet (100%) (**Table 1**).

### Aesthetics Outcomes

Esthetic assessment by the patients was performed in all groups to address scores in facial symmetry, facial scarring and facial projection. Ten patients (71.4%) reported a good aesthetic result while 4 patients referred a fair result (28.6%). A good aesthetic result was reported by 4 patients reconstructed with double-barrel flap, 4 patients reconstructed with CAD/CAM titanium mesh and 2 patients with dynamic navigation (**Table 1**).

### Dysphagia

Dysphagia was reported by the patients as “yes” or “no”. Only 2 patients reported dysphagia, while 12 patients referred normal swallowing (85.7%) (**Table 1**).

All patients were rehabilitated with an implant-supported fixed prosthesis, obtaining excellent esthetic and functional results in terms of lip competence and speech articulation. No statistically significant differences were found in prosthetic rehabilitation between the three techniques and all patients reported an intelligible speech.

## Discussion

The use of computer-assisted surgery and navigation technology in head and neck oncology was described in the early 90’s by A. Wagner (14). In recent years, the concept of “precision medicine” has become part of standard hospital practice, allowing different products to be adapted to each patient in a specific way (15). There has also been a significant development of this technology in the field of maxillofacial surgery: virtual surgical planning (VSP), CAD-CAM design and modeling and intraoperative navigation techniques have contributed, over the past few years, to simplify and improve the accuracy of surgeries. These techniques allow the pre-planning of the oncological resection, the dimensions of the neomandible and the precise location of the osteotomies in bone flaps (15, 16). CAD-CAM cutting guides help the surgical team to faithfully carry out the treatments devised, improving the precision, accuracy and reliability of the results in oncological resections and reconstructions. Intraoperative dynamic navigation systems allow immediate insertion of osseointegrated implants, contributing to faster dental rehabilitation (2, 17). Therefore, the combination of VSP and intraoperative navigation can guarantee the best possible postoperative results, especially in complex mandibular defects without occlusal stability. The main advantages of this technology are: 1) preoperative visualization of each patient’s anatomy (18); 2) it enables oncologic resection with clear margins (19); 3) it improves the accuracy of the reconstruction by simplifying the osteosynthesis of the reconstruction increasing contact surfaces and achieving a better aesthetic contour (18, 19); 4) preoperative visualization of reconstruction limitations and preventing possible complications; 5) it decreases surgical time, especially ischemia time during the free flap harvest (19); 6) it provides more predictable results, increasing the stability of the results and patient outcomes (2, 18). On the other hand, the main disadvantages of virtual surgery planning are: 1) increased costs, often due to the need for an external digital laboratory (15, 18); 2) the surgical delay involved in surgical planning and obtaining the different models and cutting guides, which can delay the beginning of treatment in oncologic patients. Although a study of the economic costs using these techniques was not carried out as it was not an objective of this study, future studies are needed to evaluate the accuracy achieved compared with standard surgery and the total value added and the cost efficiency of VSP-CAD/CAM. Overall, the decreased patient morbidity and complications and the generalized improved outcomes may potentially offset the technological costs.

The fibula flap is the technique of choice for mandibular reconstruction. One of its main disadvantages is its low height of bone to be adapted to the remaining mandible in segmental defects, resulting in a reduction of the lower facial third with the consequent aesthetic and functional sequelae (2–4). In addition, this flap allows the implantological rehabilitation of oncologic patients, although it is necessary to perform a correct distribution of the masticatory dynamic loads to avoid overloading the implants in the flap bone and the remnant mandible (2, 3). The main disadvantage of this flap is the considerable vertical discrepancy between the remnant mandible and the fibula flap, which limits the prosthetic rehabilitation of the patients due to the unfavorable crown to implant ratio (4). To solve this problem, 3D mandibular reconstruction can be complemented by using the double-barrel technique and iliac crest grafts guided by titanium mesh over the fibula flap (3). The aim of this study was to describe three different options for mandibular reconstruction and rehabilitation with dental implants in oncologic patients with segmental defects, as well as to compare the different techniques in terms of vertical bone reconstruction, peri-implant bone resorption, implant success rate, the influence of radiotherapy and the aesthetic and functional outcomes such as deglutition and swallowing.

The study shows that vertical bone augmentation is higher in patients reconstructed with double-barrel fibula flap or with iliac crest graft over the fibula. The double-barrel flap will provide a vertical dimension dependent on the thickness of the fibula bone itself, so that the bone height to be achieved can be predicted intraoperatively. This gain is greater than the amount of the vertical dimension of each segment of the fibula, since there is usually a gap between them, secondary to their adaptation to achieve an adequate ridge and basal profile of the neomandible. Besides, the iliac crest graft over the fibula presents some bone resorption. Therefore, when performing the technique, the mandibular volume is usually overcorrected. In these cases, reconstruction is less predictable although the vertical gain is similar to that achieved with the double-barrel technique.

One of the limitations of this study is the sample size. Although implant survival is favorable in all three groups, larger studies are needed to highlight significant differences. Likewise, no differences were observed between vertical bone augmentation and bone resorption between patients treated with radiotherapy and non-irradiated patients. We consider important to emphasize that this is one of the few studies that demonstrates the stability of the reconstructive results achieved with VSP, double-barrel flap, STL models, titanium CAD-CAM meshes and implants placed with intraoperative navigation in any of the three variants presented. The stability of the implant rehabilitation of the oncologic patients submitted to these techniques is also shown, with very high functional and aesthetic results.

There are very few studies comparing vertical mandibular reconstruction techniques. Navarro-Cuéllar compared in his study a series of twenty-four patients submitted to vertical reconstruction of the fibula by means of double-barrel fibula, fibula with iliac crest onlay graft and distraction osteogenesis of the fibula. The good results mainly for the first two techniques stand out, in which a vertical bone augmentation of 18.5 mm and 17.75 mm is achieved, respectively, both in patients without adjuvant treatment and in irradiated patients. In addition, bone resorption was measured and compared, and no differences were observed between these two techniques or with respect to the group of irradiated patients, showing an average bone resorption of less than 1.5mm in both groups (2). Worse results were obtained in the group treated by distraction osteogenesis, in which the mean resorption was higher than 2 mm. Yue He reported the reconstruction of seven patients with the double-barrel fibula flap with a gain of more than 30 mm. Three patients had not been irradiated and one patient received radiotherapy. However, the author provides no data on bone resorption (4). Shen published a series of forty-five cases of double-barrel fibula with implant rehabilitation in eleven of them, with good esthetic and functional results (6); however, the author did not describe the implant success rate or the peri-implant bone resorption. Ferreti described the vertical reconstruction of mandibular atrophy by means of iliac crest grafts but did not show its application in cases of segmental defect or reconstruction with peroneal flap (20). None of the works consulted compared the ratio between the width of the flap and the remaining mandible. Given the small sample size of our study, this is another of its limitations. Further studies are needed to compare the values according to anatomical variations and patient-dependent factors. Very few studies have reported comparisons of techniques for mandibular reconstruction using peroneal flap with VSP and CAD-CAM technology. Navarro-Cuéllar reported a series of eight cases in which the author demonstrated an excellent result with this technique (3). Previously, Verhoeven had reported two-dimensional reconstructions with bone resorption of up to 25% of the height provided by the iliac crest graft (21). Vermeeren described a resorption close to 50%, results similar to those obtained by Johansson (22, 23). Casap reported a case of alveolar bone augmentation by impression of a titanium shell with BMP-2/allograft with excellent results in graft ossification but with a high rate of mesh exposure (24). Roser studies the accuracy of VSP in 11 patients comparing the distance between the real mandibular osteotomies and the virtual mandibular osteotomies with an accuracy rate of 2.0 ± 1.1 mm (25). Our study demonstrates that mandibular reconstruction can be achieved safely and effectively using an iliac crest graft adapted to preformed titanium mesh using VSP and CAD-CAM technology. This mesh is optimally adapted to the peroneal flap and the patient’s anatomy. The stability of this reconstruction and the low bone resorption evaluated during the follow-up time by CBCT have also been demonstrated. The high rate of osseointegration of the implants on this bone regeneration has also been reported, providing a long-term stable occlusal rehabilitation with optimal aesthetic and functional outcomes.

The development of tissue engineering has provided the possibility to regenerate tissues from cells that develop on a matrix that guides their growth (26). Bone regeneration by means of iliac crest grafting is a form of autogenous regeneration considered to be of choice due to its osteogenic potential and its osteoinductive and osteoconductive properties (27). The success of bone grafts depends on the surfaces in contact with each other and their three-dimensional arrangement (3, 27). The design of the recipient site and its stability are also important. The fibula flap is a high quality recipient site due to its good vascularization and the provision of an extensive bone surface on which to place the graft and titanium mesh. The advantages of the iliac crest graft for 3D reconstruction of the mandible are (28): 1) high quality and quantity of cellular supply; 2) capacity for compacting the graft, which allows greater density of bone cells per unit of space; 3) good vascularization and the possibility of placing the graft in stable and well vascularized cavities. It also has certain disadvantages such as the need for a second surgical intervention, the morbidity secondary to iliac approach, the risk of exposure of the mesh containing the graft -which is higher in previously irradiated patients- and the deferred time of at least 6 months until implant surgery.

At this point and, considering the described techniques as good surgical options for vertical mandibular reconstruction, the choice of each technique will depend on the clinical status of each patient. An important point of discussion would be whether it is preferable to reconstruct the mandible in a single surgical time using a double barrel fibula flap or to delay the reconstructive process with two surgical procedures in patients in which bone regeneration is performed with titanium mesh-guided iliac crest grafting. Obviously, in patients in whom the height of the peroneal flap does not involve a high vertical discrepancy, implant rehabilitation will be faster and high accuracy can be achieved through dynamic navigation. The double-barrel fibula flap is the ideal technique for mandibular height reconstruction in order to solve the problem of bone discrepancy with the remaining mandible (2, 10). In addition, VSP, computer-aided design/computer-aided manufacturing (CAD/CAM), surgical navigation and advanced implantology complement this reconstructive technique with high precision. It has the following advantages: 1) great length of bone, allowing double-barrel reconstructions of 8-10 cm (10); 2) one surgical procedure, compared to iliac crest onlay grafts or techniques such as distraction osteogenesis (2); 3) it is a flap particularly suitable for dental implant rehabilitation and early implant placement due to the high primary stability provided by the cortical bone (12, 13); 4) this flap can be designed as an isolated bone flap or by providing a skin paddle to allow soft tissue reconstruction (2, 10); 5) it is a flap with low morbidity at the donor site; 6) it is the best surgical option for patients who will undergo radiotherapy due to the high vascularization and the minimal bone resorption of the flap (29); moreover, in cases of implant rehabilitation, low peri-implant resorption has been demonstrated. The major disadvantage of this technique is the limitation in patients with defects larger than 12-14 cm, which would require a flap length of almost 25 cm, which would increase the morbidity of the donor site (29) and decrease the length of the vascular pedicle for microvascular anastomosis. In these cases, an alternative surgical option is to reconstruct with the double-barrel only the mandibular region compromised with the occlusion in which the dental implants will be placed. Therefore, in oncologic patients, especially those receiving radiotherapy and, in patients with segmental defects smaller than 12-14 cm, this is the technique of choice. It allows early prosthetic rehabilitation and high success rate of the implants (2, 12, 13).

The cortico-cancellous iliac crest graft allows deferred mandibular reconstruction. It presents low bone resorption and high success rate of dental implants. It allows an adequate vertical reconstruction of the mandible, solving the problem of discrepancy with the remaining mandible and provides high volumes of autologous bone that supports well the treatment with radiotherapy (20). However, it has a number of disadvantages: it requires a second surgical procedure; it is essential to overcorrect the area to be treated by approximately 25% to compensate for the loss due to bone resorption, which entails the need for remodeling in some cases (30); the donor area implicates certain morbidity (6); it is necessary to wait at least six months for the ossification of the graft before placing dental implants, thus prolonging the prosthetic rehabilitation time (9). This technique is recommended mainly in patients who are not going to receive radiotherapy with extensive defects in the symphysis and mandibular body in which a double-barrel flap is not possible (2). When harvesting this technique, it is important to perform a cervical approach for the placement of the graft on the peroneal flap to avoid its contamination by flora of the oral cavity (1, 2). Implant success rates and peri-implant bone resorption are similar to those presented by the double bar fibula flap; however, the need for several surgical procedures and the impossibility of receiving radiation while the titanium mesh maintains the graft until ossification, limit the use of this technique, remaining as a second option in oncologic patients. Titanium mesh is ideal for the execution of this technique. It is a biocompatible material, with a high capacity to prevent deformation, and a 3D stability and adaptation that prevents the graft from collapsing. It is a non-absorbable material and protects the graft from invasion by adjacent soft tissue. It provides a smooth surface that isolates the graft from bacterial colonization and the elasticity of titanium prevents compression of the graft by intraoral tissues and mobile elements. In addition, the micropores on its surface maintain the vascular supply to the recipient area. Several studies have reported mesh exposure and bone resorption (31, 32); however, in our series we have demonstrated maintenance of the mesh without exposure in the oral cavity and minimal bone resorption due to the virtual surgical planning and the cervical approach performed to avoid contact between the graft and the oral cavity. Conventional surgery for titanium mesh with bone graft may show inadequate adaptation, irregular edges that may lead to exposure of the mesh and dead spaces that result in early bone resorption and the development of complications such as infection. Optimal mesh adaptation and proper design achieved by VSP and STL model production are critical for good aesthetic and functional results. Thus, it is possible to recreate and maintain the shape of the previous mandible, allowing a correct maxillo-mandibular position and a more predictable implant placement. The major limitation of VSP is the added cost of the surgical procedure; in any case, the notorious improvement of the results obtained needs to be valued with respect to the cost of the technology. Since this is one of the few works that report the experience with the use of this technology for mandibular reconstruction in oncologic patients, more studies are needed to evaluate and compare the results obtained with surgery performed using VSP, CAD-CAM technology, STL models and intraoperative navigation with respect to conventional surgery.

Although it is not the subject of this study, several authors advise against osteogenic distraction of the fibula in oncologic patients with a potential need for adjuvant radiotherapy treatment because of the 3-5 month period until stable vertical gain is achieved and, above all, because of the risk of exposure of the distractor and the bone during radiotherapy treatment. In addition, the technique requires three surgical procedures since the reconstruction until the distractor removal and implant placement (2).

In house navigation of dental implants allows real-time anatomical assessment of the patient’s neo-mandible on which they are to be inserted. While VSP and CAD-CAM technology allow a more predictable flap design and mandibular reconstruction, it also provides the advantage of knowing the limitations of implant placement in an anatomically complex site (33). Thus, in patients rehabilitated with this technique, the VSP made possible to predict the exact position of the osteosynthesis material in the neomandible, proceeding to total or partial removal if it interfered with the correct position of the implant for a correct occlusal rehabilitation. The titanium plates with high adaptation to the anatomy of the flap used for the reconstruction may lead, in many cases, to place the implants to avoid interference with osteosynthesis screws. Intraoperative navigation allows in these cases to redirect the position of the implant and to achieve predictable, more accurate results with a reduction in surgical time. We used the in-house navigation technique for patients without dental implants in the previous fibula flap. Nowadays, and due to VSP, it is possible to perform mandibular reconstruction both in double-barrel or single fibula with immediate implant placement without the need of a second surgical time for implant placement through dynamic navigation. Therefore, we recommend this technique in patients in whom implant placement has not been performed previously.

From the aesthetic and functional point of view, mandibular reconstruction according to its original dimensions will provide a good projection of the lower third of the face. Otherwise, tissue collapse will occur, resulting in aesthetic and functional sequelae. Another point of discussion would be whether it is necessary to place the fibula at the inferior border of the mandible or in the midbody proximal to the alveolar ridge of the remnant mandible. Although the fibula positioned in the upper part of the defect is widely described in the literature, our recommendation would be to reconstruct the inferior border of the mandible to achieve optimal facial symmetry with a harmonious facial profile and to perform complementary techniques such as the double-barrel fibula flap to restore the previous height of the mandible and to place immediate implants. The reconstruction of the mandible by means of VSP, CAD-CAM and STL models makes possible to restore bone volumes similar to the previous dimensions, leading to a harmonious physical appearance of the patients. Mandibular profiles similar to the native mandible are achieved and osteosynthesis material arrangements that could compromise esthetics are avoided. In addition, functional results such as lip competence, deglutition and speech articulation are excellent in the patients of our study. In this study, the authors evaluated the functional outcomes according to patients’ oral feeding ability. Patients were classified according to their ability to develop an unrestricted diet or a soft/liquid diet. Only 2 patients referred dysphagia and most of the patients reported a normal diet, and masticatory function was preserved after mandibular reconstruction with the described techniques. Similarly, 71.4% of the patients obtained good esthetic results both objectively and from the subjective evaluation of each patient.

It should be noted that segmental mandibular defects secondary to oncologic processes reconstructed with peroneal flap can solve the problem of vertical discrepancy and three-dimensional volume of the deficient mandible by means of double barrel techniques or by cortico-cancellous iliac crest grafting with titanium mesh developed by VSP, STL models and CAD-CAM technologies. In all cases the reconstruction can be completed by implant rehabilitation and intraoperative navigation. These techniques allow a precise 3D reconstruction and can be combined according to the needs of each patient, obtaining excellent esthetic and functional results.

Finally, it is important to emphasize that it is necessary to acknowledge all these surgical techniques, in order to individualize the mandibular reconstruction in oncologic patients. Multi-stage implementation of virtual surgical planning (VSP) with the use of stereolithographic models (STL), 3D printing of patient-specific surgical guides, CAD/CAM titanium mesh and intraoperative implant dynamic navigation for 3D mandibular defects offer a reconstructive accuracy improving the operative efficiency, reducing the complication rate and enabling the comprehensive rehabilitation of the patients, providing aesthetic and functional results that return quality of life to oncologic patients.

## Data Availability Statement

The original contributions presented in the study are included in the article/supplementary material. Further inquiries can be directed to the corresponding author.

## Ethics Statement

Written informed consent was obtained from the individual(s) for the publication of any potentially identifiable images or data included in this article.

## Author Contributions

Conceptualization: RA-C and CN. Methodology: RA-C, MT, and JS. Software: AD-M and ÁS. Validation: RA-C, CN, and JS. Formal analysis: SO and DG. Investigation: RA-C and AD-M. Resources: IN, CN, MA, and GA. Data curation: JS and IN. Writing—original draft preparation: RA-C. Writing—review and editing: RA-C and CN. Visualization: SO. Supervision: RA-C. Project administration: RA-C and JS. All authors contributed to the article and approved the submitted version.

## Funding

This study was supported by a grant from Ticare (R) implants (Mozo-Grau, Valladolid, Spain). The funder was not involved in the study design, collection, analysis, interpretation of data, the writing of this article or the decision to submit it for publication.

## Conflict of Interest

The authors declare that the research was conducted in the absence of any commercial or financial relationships that could be construed as a potential conflict of interest.

## Publisher’s Note

All claims expressed in this article are solely those of the authors and do not necessarily represent those of their affiliated organizations, or those of the publisher, the editors and the reviewers. Any product that may be evaluated in this article, or claim that may be made by its manufacturer, is not guaranteed or endorsed by the publisher.
